# Utility of metabolic ratios in the diagnosis of tumor Thrombus on F-18 FDG PET/CT

**DOI:** 10.1186/s41824-024-00201-z

**Published:** 2024-05-08

**Authors:** Deepanksha Datta, Rajesh Kumar, Akhil Dhanesh Goel

**Affiliations:** 1 Department of Nuclear Medicine, All India Institute of Medical Sciences, Jodhpur, India; 2 Department of Community Medicine, All India Institute of Medical Sciences, Jodhpur, India

**Keywords:** Tumor thrombus, F-18 FDG PET/CT, Metabolic ratios, Quantitative analysis

## Abstract

**Background:**

This study aims to predict quantitative parameter in form of metabolic ratios to diagnose tumor thrombus on F-18 FDG PET/CT.

**Methods:**

This is a retrospective study from Nuclear Medicine department at All India Institute of Medical Sciences, Jodhpur, India. Patients with malignancies who underwent F-18 FDG PET/CT in our department or images sent for review from February 2020 till September 2022 were screened for tumor thrombus that comprised study group. Control group had patients with malignancy and no imaging evidence of tumor thrombus. Metabolic activities (SUVmax) of tumor thrombus, liver and descending aorta in study group, and that of IVC, liver and descending aorta in control group were recorded. Metabolic ratios of tumor thrombus to liver (SUR L) and to aorta (SUR A) in study group, and IVC to liver (SUR* L) and to aorta (SUR*A) in control group were compared using receiver operator curves.

**Results:**

Of 2277 studies screened, 12 had tumor thrombus. The most common primary malignant site and vessel involved were lung and IVC respectively. The median (IQR) SUR L, SUR A, SUR* L and SUR* A were 2.5 (3.25), 2.6  (6), 0.67 (0.18) and 1 (0.17) respectively. Area under ROC for SUR L and SUR A were 0.983 [95% CI: 0.955–1.0] and 0.958 [95% CI: 0.90–1.0] respectively. The ideal cut-off for SUR L was 0.953 (sensitivity 92.3%, specificity 98.0%) and for SUR A was 1.42 (sensitivity 84.6%, specificity 98.0%).

**Conclusion:**

Metabolic ratios of tumor thrombus to liver (SUR L) and aorta (SUR A) have good diagnostic performance and can be useful in studies with non-iodinated contrast CT.

## Introduction

Tumor thrombus (TT) is a rare but serious complication of any malignancy. Its presence has a negative impact on the staging, management and prognosis of any primary malignancy (Quencer et al. 2017). A tumor thrombus is usually differentiated from a bland thrombus by the presence of an expansile intravascular mass with contrast enhancement on contrast-enhanced computed tomography (CECT) or contrast-enhanced magnetic resonance imaging (CE – MRI), and with intravascular FDG uptake on positron emission tomography (FDG PET/CT) (LeGout et al. 2020). Although the presence of intravascular FDG uptake is highly sensitive for the identification of TT on PET/CT (Kikuchi et al. 2004; Lai et al. 2007; Davidson et al. 2009; Kaida et al. 2007; Tateishi et al. 2005; Kurtovic et al. 2005; Sharma et al. 2012; Aurangabadkar et al. 2013), it is usually just a visual analysis and a few false positives have been reported (Kikuchi et al. 2004). We undertook this study with the aim to analyse the metabolic uptake of TT in terms of metabolic ratios, and have attempted to define a criteria to diagnose TT on FDG PET/CT in a known case of malignancy.

## Materials and methods

### Study setting and patient criteria

This was a retrospective study conducted in the Department of Nuclear Medicine at All India Institute of Medical Sciences, Jodhpur (India), a tertiary care referral university hospital in North India. This study was approved by the the Institute Ethics Committee (IEC No. AIIMS/IEC/2023/4333 dated 6th March 2023). All F-18 FDG PET/CT scans of patients with biopsy proven malignancy, which were either performed in department from February 2021 to 2023, were screened for tumor thrombus. Presence of intravascular FDG uptake with contrast enahncemnet and/or vessel expansion was considered positive for tumor thrombus, and included in the study group. The control group included 50 random patients with known malignancy and underwent F-18 FDG PET/CT at our department, and did not have imaging evidence of tumor thrombus.

### Acquisition of 18-FDG PET/CT

F-18 FDG PET/CT imaging was carried out in accordance with the standard clinical PET protocol as per the EANM guidelines (Boellaard et al. 2015). The patients were intravenously injected with F-18 FDG 3.7 MBq/kg body weight to a maximum dose of 370 MBq after a 4–6 h fasting period. All patients were imaged with an integrated PETCT system (DiscoveryGE MIDR710). After 45–60 min of uptake period at rest, in a dimly lit quiet room, the images were acquired at 1 min per bed position. In the patients with serum creatininne under normal limits and with no other contraindications to iodinated contrast, the PET scan was acquired together with the CECT scan, a delay of 70 s was between the intravenous iodinated contrast injection and aqcuistion of CT scan.

Presence of intra-vascular FDG uptake (focal or diffuse) with associated CT features was considered positive for tumor thrombus (LeGout et al. 2020), and served as the only inclusion criteria in the study group. The findings on PET/CT were independently verified by two Nuclear Medicine physicians each with more than 5 years of experience. Those scans with either one disagreement were exlcuded from the study group.

### Definition and calculation of metabolic ratios of tumor thrombus

In the study group, we recorded the metabolic activities of the tumor thrombus (SUV T), patient’s liver (SUV L) and descending thoracic aorta (reference for blood pool, SUV A) in terms of SUVmax (Standardised uptake value; g/ml) normalized to lean body mass. The volumes of interest (VOI) for liver was drawn on caudate lobe, and for descending thoracic aorta was drawn as just above the diaphragm. The biochemical and clnincal profiles for all the patients included in the study and control groups were retrospectively screened and were negative for any liver abnormalities, intravsaulcular interventions and vasculitis. The metabolic ratios of tumor thrombus to liver and tumor thrombus to descending aorta were calculated using the formulae,

$$Metabolic Ratio Of Tumor Thrombus To Liver (SUR L) = (SUV T)/ (SUV L) $$

$$\eqalign{ Metabolic{\rm{ }}Ratio{\rm{ }}Of{\rm{ }}Tumor{\rm{ }}Thrombus{\rm{ }}To{\rm{ }}Descending{\rm{ }}Aorta{\rm{ }}\left( {SUR{\rm{ }}A} \right) \cr & = \left( {SUV{\rm{ }}T} \right)/{\rm{ }}\left( {SUV{\rm{ }}A} \right) \cr}$$

In the control group, the metabolic activity of IVC (SUV IVC), patient’s liver (SUV* L) and descending thoracic aorta (reference for blood pool, SUV*A) in terms of SUVmax (Standardised uptake value; g/ml) normalized to lean body mass. IVC was considered as a control for the tumor thrombus because most of the tumor thrombi are venous thrombi, IVC is the largest vein in the body, and IVC is the most commonly reported vessel involved by the tumor thrombus in most of the malignancies (Sharma et al. 2011; Ravina et al. 2014).

The metabolic ratios in the control group were thus,

$$Metabolic RatioOfIVCToLiver(SUR?L)=(SUVT)/(SUV?L)$$

$$MetabolicRatioOfIVCToDescendingAorta(SUR?A)=(SUVIVC)/(SUV?A)$$

The metabolic ratios with liver and descending aorta were compared in the study and control groups (SUR L vs. SUR* L) and (SUR A vs. SUR* A).

### Statistical analysis

Continuous data were expressed as median with inter-quartile range (IQR). The diagnostic performance of the metabolic ratios were calculated using receiver-operator characteristics (ROC) analysis and area under the curve (AUC). Data analysis was done using the software Statistical Package for Social Studies ver 23 (IBM Technologies, USA).

## Results

**Fig. 1:**
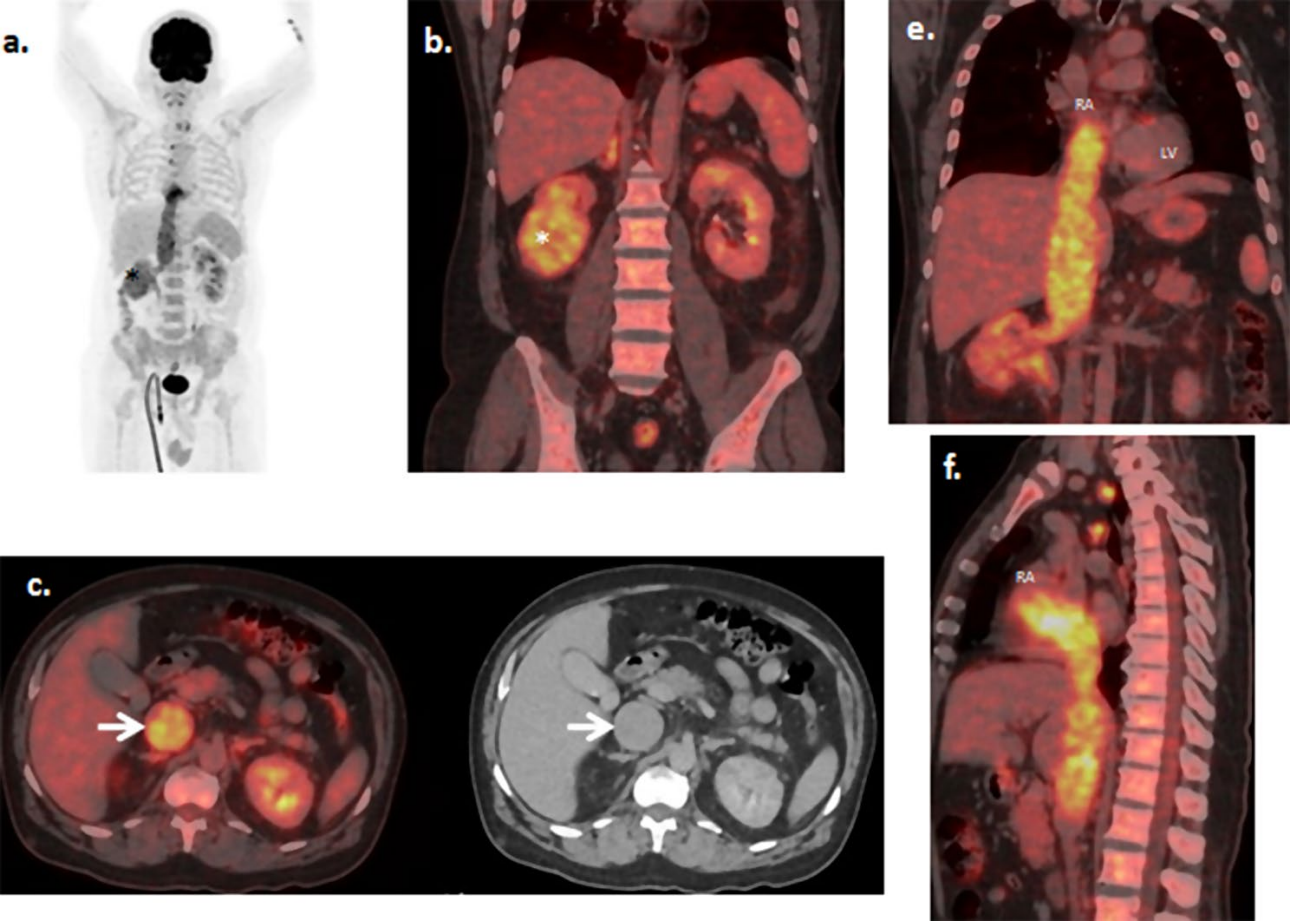
A 49 year gentleman, recent diagnosed case of right renal carcinoma, underwent PET/CT for staging by intravenous injection of 7mCi of F-18 FDG. Maximum Intensity Projection image (MIP, **a**) and sagittal fused PET/CT image (**b**) show metabolically active right renal mass in its lower pole (*asterisk*). Fused and CT axial images (**c**) show diffuse intravascular FDG uptake in right renal vein and dilated IVC suggestive of tumor thrombus (*white arrows*). The fused coronal (**e**) and sagittal (**f**) images show the tumor thrombus reaching upto right atrium

Of 2277 FDG PET/CT studies screened, 12 had tumor thrombus and were included in the study group. The most common primary site of malignancy and vessel involved were lung and IVC respectively. The patient characteristics are shown in (Table 1). Figure 1 shows a pictorial representation of a tumor thrombus from right kidney reaching upto right atrium.

**Table 1:** Baseline characteristics and disease parameters in patients with tumor thrombus

Characteristic		Number
Total patients of malignancy screened		2277
Total patients with TT		12/2277
Median Age in years (range)		56.5 (8–74)
Gender Male (percent)		6 (50)
*Site of Primary Malignancy in patients with TT (n = 12)*		
	Lung (percent)	3/12 (25)
	Renal (percent)	2/12(17)
	Liver (percent)	2/12(17)
	Bone (percent)	2/12(17)
	Lymphoma (percent)	2/12(17)
	Thyroid (percent)	1/12 (8)
*Vessels Involved by TT (n = 19)*		
	IVC(percent)	5/19 (26)
	Iliac veins (percent)	5/19 (26)
	SVC (percent)	3/19 (16)
	Brachiocephalic vein (percent)	2/19 (11)
	Renal (percent)	1/19 (5)
	IJV(percent)	1/19 (5)
	Pulmonary vein (percent)	1/19 (5)
	Portal vein (percent)	1/19 (5)

*TT – tumor thrombus, IVC – inferior vena cava, SVC – superior vena cava, IJV – internal jugular vein

The median (IQR) SUR L and SUR A were 2.5 (3.25) and 2.6 (6) respectively (Table 2). In the control group, the median (IQR) SUR* L and SUR*A were 0.67 (0.18) and 1 (0.17) respectively.

**Table 2:** Metabolic parameters of tumor thrombus group and control group

Sl. No.	Study Group, *n* = 12		Control Group, *n* = 50	
1	Median SUVmax of TT (IQR)	5.1 (7.2)	Median SUVmax of IVC	2.0 (0.8)
2	Median SUVmax Liver	2.0 (1.2)	Median SUVmax *Liver	3.0 (1.0)
3	Median SUVmax A	1.6(0.6)	Median SUVmax *A	2.0 (0.6)
4	Median SUR L	2.5 (3.25)	Median SUR* L	0.67(0.18)
5	Median SUR A	2.6(6.0)	Median SUR* A	1(0.17)

*TT – Tumor thrombus, IVC – inferior vena cava, A – Descending Thoracic Aorta, SUR L - Metabolic ratio of Tumor thrombus to liver, SUR A –Metabolic ratio of Tumor thrombus to descending thoracic aorta, SUR* L - Metabolic ratio of IVC to liver, SUR* A - Metabolic ratio of IVC to descending thoracic aorta

**Values are expressed as median (Interquartile range, IQR)

**Fig. 2:**
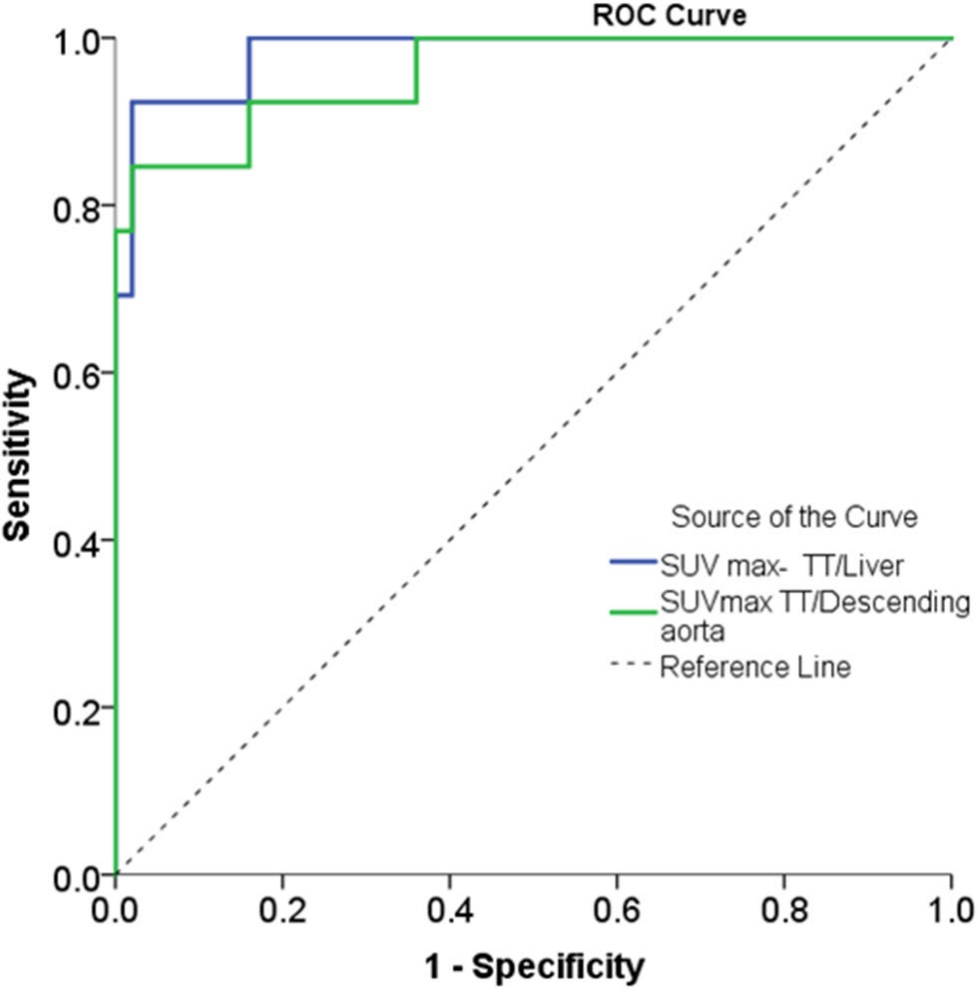
The Receiver Operator Curve (ROC) analysis and Area Under the Curve (AUC)

Area under the ROC curve for SUR L was 0.983 [95% CI: 0.955–1.0] and SUR A was 0.958 [95% CI: 0.90–1.0] (Fig. 2). The ideal cut-off for SUR Lwas 0.953 (sensitivity − 92.3%, specificity-98.0%), and for SUR Awas 1.42 (sensitivity − 84.6%, specificity 98.0%).

## Discussion

The diagnosis of tumor thrombus is usually based on the presence of FDG uptake inside the vessel, i.e. intraluminal FDG uptake. This method of identification is by visual analysis, and can be dependent on nuclear medicine reader. Few false positives have been reported that show increased FDG uptake inside the vessels with absence of enhancement, like septic and aseptic venous venous thrombi, and intravenous catheters (Kikuchi et al. 2004; Miceli et al. 2004; Do et al. 2006; Rondina et al. 2012) Also increased blood pool activity due to patient, injection or attenuation related causes can result in false diagnosis of tumor thrombus. This study was designed to develop quantitative parameters that can aid in early and accurate diagnosis of tumor thrombus, and can be later used to differentiate it from bland thrombus or veno-thrombo embolus (VTE).

Sharma et al. proposed SUV max cut off of 3.63 (sensitivity − 71.4%, specificity- 90%) to differtiate tumor thrombus from bland thrombus (Sharma et al. 2011). In our study, we analysed the metabolic ratios of tumor thrombus with the liver and descending aorta on FDG PET/CT, and compared them with the same ratios in the control group. In our study IVC was the most common vessel involved, which is also reported to be commonly involved by tumor thrombus in the available literature (Sharma et al. 2011; Ravina et al. 2014). Thus in the control group, IVC was taken as the reference vessel, and its intravascular FDG uptake was compared with liver and descending aorta. On comparison, the areas under the curves of both SUR L (0.983, 95% CI: 0.955-1.0) and SUR A (0.958, 95% CI: 0.90-1.0) of study groups were high, the value of SUR L was higher than that of SUR A, however there was no statitiscal difference between them.

The ideal cut off of SUR Lwas 0.953 (sensitivity: 92.3%, specificity : 98%) and of SUR Awas 1.42 (sensitivity : 84.6% specificity : 98%). This shows intravascular FDG uptake similar to that of liver has a high suspicion for tumor thrombus. Although the tumor thrombus shows FDG uptake more than that of the descending aorta (blood pool), its metabolic activity should be compared with the liver owing to lower value of its metabolic ratio with descending aorta.

Till now there is no consensus in determining a cut off of SUV max for diagnosis of tumor thrombus or differentiatingit from the bland thrombus. This study emphasise that metabolic ratios can serve as an important diagnostic tool for accurate diagnosis of tumor thrombus even in the absence of iodinated contrast media. This study can serve as a basis for further prospective studies on quantitative analysis of tumor thrombus.

## Conclusion

Metabolic ratios of tumor thrombus with liver and descending aorta have a good diagnostic performance in diagnosis of tumor thrombus. These parameters can be useful in non-iodinated contrast PET/CT studies.

## Data Availability

The data set generated and analysed during the current study are avalaible with the corresponding author on request.

## Abbreviations

TT: Tumor Thrombus
F-18 FDG: F-18 2-fluoro 2-deoxy D glucose
PET/CT: Positron Emission Tomography
EANM: European Association of Nuclear Medicine
CECT: Contrast Enhanced Computed Tomography
CT: Computed Tomography
CE-MRI: Contrast enhanced Magnetic Resonance Imaging
IVC: Inferior Vena Cava
SUV T: SUVmax of Tumor Thrombus
SUV L: SUVmax of Liver in study group
SUV A: SUVmax of descending thoracic aorta in study group
SUV IVC: SUVmax of IVC in control group
SUV* L: SUVmax of liver in control group
SUV*A: SUVmax of descending thoracic aorta in control group
SUR_L_: Metabolic ratio of tumor thrombus to liver
SUR_A_: Metabolic ratio of tumor thrombus to descending thoracic aorta
SUR^*^_L_: Metabolic ratio of IVC to liver
SUR^*^_A_: Metabolic ratio of IVC to descending thoracic aorta
IQR: Inter-quartile range
ROC: Receiever operator curve
